# α-Silicene as oxidation-resistant ultra-thin coating material

**DOI:** 10.3762/bjnano.8.182

**Published:** 2017-08-31

**Authors:** Ali Kandemir, Fadil Iyikanat, Cihan Bacaksiz, Hasan Sahin

**Affiliations:** 1 Department of Materials Science and Engineering, Izmir Institute of Technology, 35430, Izmir, Turkey; 2 Department of Physics, Izmir Institute of Technology, 35430, Izmir, Turkey; 3 Department of Photonics, Izmir Institute of Technology, 35430, Izmir, Turkey; 4 ICTP-ECAR Eurasian Center for Advanced Research, Izmir Institute of Technology, 35430, Izmir, Turkey

**Keywords:** coating material, density functional theory, oxidization, silicene, silver

## Abstract

By performing density functional theory (DFT)-based calculations, the performance of α-silicene as oxidation-resistant coating on Ag(111) surface is investigated. First of all, it is shown that the Ag(111) surface is quite reactive against O atoms and O_2_ molecules. It is known that when single-layer silicene is formed on the Ag(111) surface, the 3 × 3-reconstructed phase, α-silicene, is the ground state. Our investigation reveals that as a coating layer, α-silicene (i) strongly absorbs single O atoms and (ii) absorbs O_2_ molecules by breaking the strong O–O bond. (iii) Even the hollow sites, which are found to be most favorable penetration path for oxygens, serves as high-energy oxidation barrier, and (iv) α-silicene becomes more protective and less permeable in the presence of absorbed O atom. It appears that single-layer silicene is a quite promising material for ultra-thin oxidation-protective coating applications.

## Introduction

Surface protection against degradation of a material due to a reaction with its environment has attracted intensive attention of researchers for decades. In order to prevent the loss of important properties (such as conductivity, reflectivity, and mechanical and thermal resistance) of a material, surface protection has been vital nearly for all application areas. As a well-known mechanism of electrochemical corrosion, the formation of rust is an un-solicited reaction between a metal and oxygen. For that reason, protection of surfaces from oxygen has become an important field. Although macroscale and microscale coatings have been used intensively in surface protection for a long time [1–2], two-dimensional (2D) materials have become new candidates for nanoscale coatings for different material groups. Therefore, coating mechanisms at the nanoscale take are of high interest in nanotechnology, and new candidates for nanostructural protection are needed to be understood in detail.

Due to its extraordinary structural and electrical properties, graphene as 2D material has garnered huge interest in nearly all science branches [3–4]. Because of the high impermeability, graphene has also been thought as a corrosion-protection barrier [5–7]. Kirkland et al. investigated the electrochemical response of graphene-coated metal surfaces and found that graphene causes reduction in the corrosion rate [8]. In addition, Topsakal et al. showed that graphene is a suitable coating material to protect surfaces from oxidation by performing DFT-based calculations [9]. Bulk forms of transition-metal dichalcogenides (TMDs) are well-known coating materials, and the respective 2D TMDs can be used as surface protection. In addition, MoS_2_ is one of the most widely used lubricant coating material [10]. Theoretical and experimental studies have demonstrated that single-layer MoS_2_ and single layer W(S/Se)_2_ can be used as a protective nanocoating material [11–14].

One of the most challenging members of the 2D material family is silicene [15–19], the silicon analogue of graphene. After theoretical prediction [18], silicene was synthesized [19] on a silver surface in the 3 × 3-reconstructed α-form. Differing from graphene, silicene exhibits low buckling in which atoms in the different sub-lattices are shifted oppositely in the out-of-plane direction. The buckled structure of silicene forms perfect sites to capture oxygen atoms. It is known that silicon atoms tend to bond oxygen atoms strongly and form various stable oxidized silicon structures. Therefore, silicene can be a potential coating material for protection at the nanoscale.

On the other hand, silver is being used in our daily life in jewelry, silverware, decorative objects and electronics. Although the oxidation of silver forms thin layer of Ag_2_O, which protects from more oxygen diffusion into silver surface, it is an undesirable reaction. Previous studies have shown that oxidation of silver surfaces leads to increase in work function, color change and significant deterioration of surface quality [20–21]. For that reason, coating of silver for protection to oxidation is needed.

In this study, we examined the coating performance of α-silicene against oxidation on the most preferable substrate metal for silicene, silver. In first step, we studied the adsorption of an oxygen atom and an oxygen molecule on bare silver with 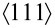 growth direction because silicene has been synthesized on Ag(111) substrates [19]. Then, the adsorption of the oxygen atom/molecule on α-silicene over Ag(111) was investigated. It was shown that α-silicene is quite reactive regarding oxidation. In addition, we focused on oxidation scenario of silver in the presence of α-silicene. A large energy barrier for oxidation was obtained by performing indentation calculations. In conclusion, it was found silicene exhibits good performance in the protection of a Ag(111) surface against oxidation.

## Results and Discussion

### Computational methodology

To obtain the preferable crystal structure of α-silicene on Ag(111), four-layer Ag(111) composed of two fixed bottom layers and two free upper layers, was prepared as supercell structure with dimensions of 4 × 4 × 1. Thus, the surface of silver successfully simulated with and without silicene on top of Ag(111). First principles calculations were performed using the Vienna ab initio simulation package (VASP) [22–23], which is based on density functional theory. The projector-augmented wave (PAW) [24–25] formalism was used in the calculations. For the exchange–correlation energy, the generalized gradient approximation of the Perdew–Burke–Ernzerhof (GGA-PBE) [26] functional was used in conjunction with a semi-empirical scheme for including van der Waals (vdW) interaction dispersive forces developed by Grimme [27]. The structural relaxations were performed with a plane wave cut-off energy of at 500 eV. A 3 × 3 × 1 *k*-point mesh was used for the structural relaxation. The criterion of convergence of energy was chosen as 10^−5^ eV between two ionic steps, and the maximum force allowed on each atom is 0.1 meV/Å. At least 13 Å of vacuum were applied along *z*-direction to hinder interactions between the adjacent cells.

### Oxidation of the bare Ag surface

**Figure 1:**
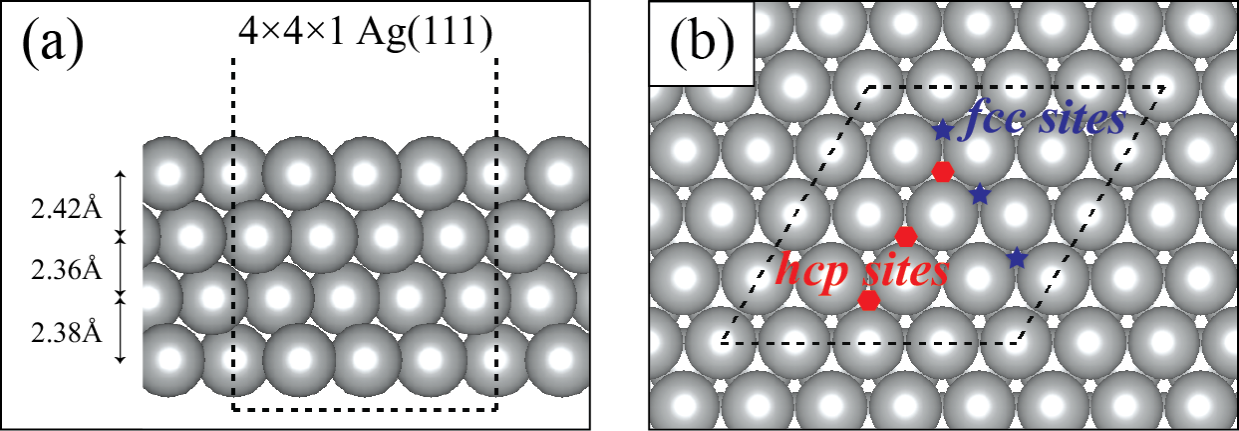
(a) Side view of the Ag(111) supercell structure and (b) top view of the Ag(111) supercell structure with possible oxygen captured sites. Definitions and oxygen binding energies of all sites are given in Table 1.

Because the buckled structure of silicene grows sleekly on a silver surface, one may expect unique and enhanced coating performance against oxidation. As shown in Figure 1, we first investigate how strongly an oxygen atom and an oxygen molecule interact with possible sites on the Ag(111) surface. Two sites were found to be preferable locations for oxygen atoms on the Ag(111) surface, the hcp and fcc hollow sites. These sites are shown in Figure 1b.

**Figure 2:**
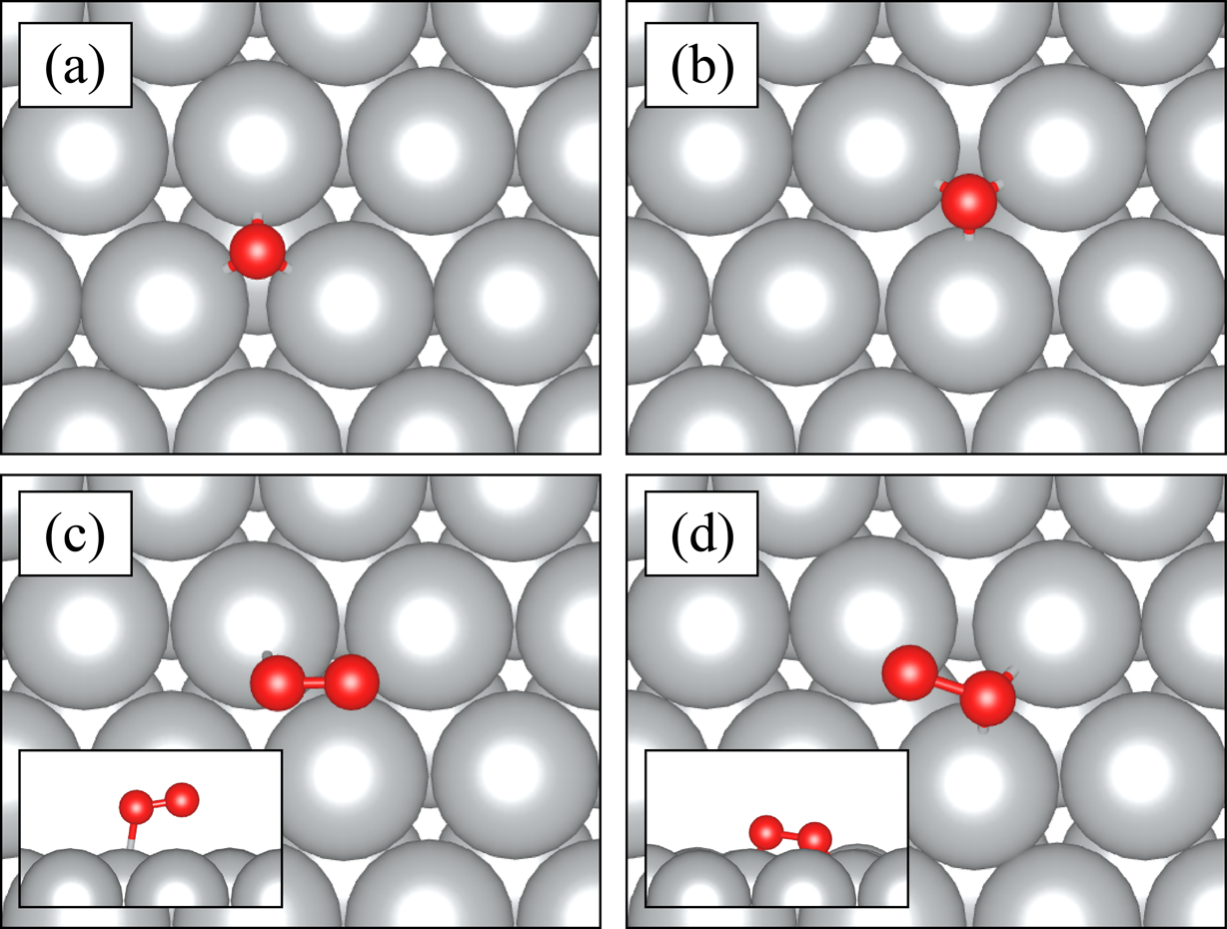
Top view of Ag(111) geometric structures after capturing an oxygen atom and an O_2_ molecule. (a) hcp hollow site and (b) fcc hollow site for oxygen, (c) magnetic and (d) nonmagnetic O_2_ molecule adsorption on Ag(111). The insets in panel (c) and panel (d) show the bonding characteristics of O_2_ molecule.

Figure 2a and Figure 2b show how an oxygen atom is adsorbed at the silver surface at the hcp and fcc hollow sites, respectively. The binding energy of single oxygen on the Ag(111) surface is about 3.9 eV. The energy difference between sites is only 110 meV. It is seen that silver surface strongly captures single oxygen atoms. Oxygen in fcc site forms a bond distance with neighbor silver atoms of about 2.14 Å. The presence of oxygen causes a distortion of about 9.8% and pushes neighbor atoms in this site. Although oxygen at a hcp site has the same bond distance with neighbor atoms as at the fcc site, the presence of oxygen causes distortion of about 8.9% at the hcp site. It appears that, absence of the lower silver atom at the fcc site results in a deeper penetration of the oxygen atom than at the hcp site.

Compared to a single oxygen atom, the O_2_ molecule behaves differently on a bare silver surface. Figure 2c and Figure 2d) show how the O_2_ molecule interacts with the silver surface. Magnetic and nonmagnetic states of O_2_ molecule are observed while oxygen is captured at the silver surface. In the magnetic state, only one of the oxygens come closer to the surface. In the other case, both oxygen atoms come closer to the surface. In both cases, the binding energies are ca. 200 meV. Therefore, it is seen that O_2_ molecule tends to bind to the silver surface. In the magnetic state, the oxygen–oxygen distance is around 1.26 Å, which is close to oxygen–oxygen distance in an oxygen molecule in the vacuum state. In the nonmagnetic state, the oxygen–oxygen distance is around 1.41 Å. The silver surface weakens the oxygen–oxygen bond in the nonmagnetic system. In addition, the distance between oxygen and silver is nearly the same as the distance between a single oxygen atom and silver distance, which is 2.14 Å. Similar to adsorption of a single atom, the oxygen molecule distorts silver surface at that site. Therefore, the oxygen–oxygen bond can be broken through a thermally induced process and the silver surface can exhibit oxygen atoms.

### Oxidation of the silicene-coated Ag surface

**Figure 3:**
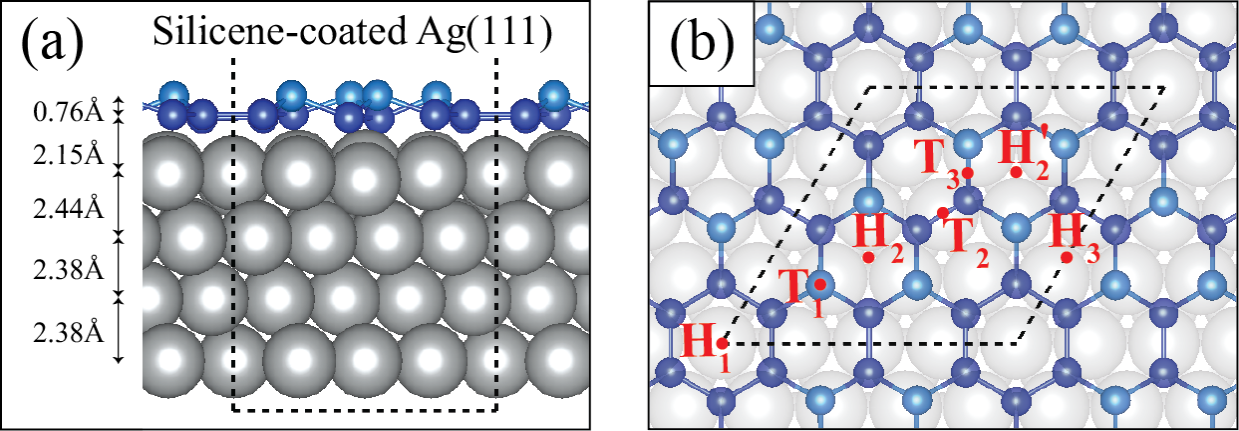
(a) Side view of the silicene-coated Ag(111) supercell structure and (b) top view of the silicene-coated Ag(111) supercell structure with possible oxygen-capture sites. Definitions and oxygen binding energies of all sites are given in Table 1.

In this section, the silicene-coated Ag(111) surface is investigated. We consider the experimentally realized structure of silicene on Ag(111), α-silicene [19]. Albeit with a different notation, it was also shown that α-silicene is the thermodynamically favorable phase under a wide range of conditions [17]. Differing from the theoretical predicted simply buckled silicene, α-silicene has a 3 × 3-reconstruction in which six silicon atoms form a sub-layer over the other twelve silicon atoms in the supercell as shown in Figure 3. Blue and turquoise atoms represent the lower and upper Si atoms respectively. In the presence of silicene, the interlayer distance of the uppermost Ag layers are slightly changing and the distance between Ag surface and silicene is found to be ca. 2.15 Å.

In Figure 3b, possible sites for oxygen on silicene-coated Ag(111) are demonstrated. There are six possible sites due to symmetry in silicene-coated Ag(111). These sites are named according to hollow and top sites of neighboring silicon atoms in the buckled silicene structure. H*_x_*s are for hollow sites, T*_x_*s are for top sites. The definition of sites can be found in Table 1 in detail. These six possible sites reflect all possible final configurations in the system. Table 1 shows the oxygen binding energy of all possible sites for silicene-coated Ag(111).

**Table 1:** Binding energies of O/O_2_ on Ag(111) and silicene-coated Ag(111). Ag refers to the Ag(111) surface, Si/Ag refers to silicene-coated Ag.

system	site	name	binding energy (eV)	Δ_BE_ (meV)

O@Ag	fcc	cubic close-packed hollow site	3.90	—
	hcp	hexagonal close-packed hollow site	3.79	110
O@Si/Ag	H_1_	site between six lower silicon atoms	6.45	70
	T_1_	top site of upper silicon atom	5.78	740
	H_2_	site between three upper and three lower silicon atoms	6.00	520
	T_2_	top site between two lower silicon atoms	6.27	250
	T_3_	top site between one upper and one lower silicon atom	6.52	—
	H_3_	site between four lower and two upper silicon atoms	5.84	680
O_2_@Ag	mag	magnetic O_2_ molecule on the surface	0.21	—
	n-mag	non-magnetic O_2_ molecule on the surface	0.19	20
O_2_@Si/Ag	in-S	O_2_ molecule inside silicene	6.70	—
	top-S	O_2_ molecule on top of silicene	2.06	4640

**Figure 4:**
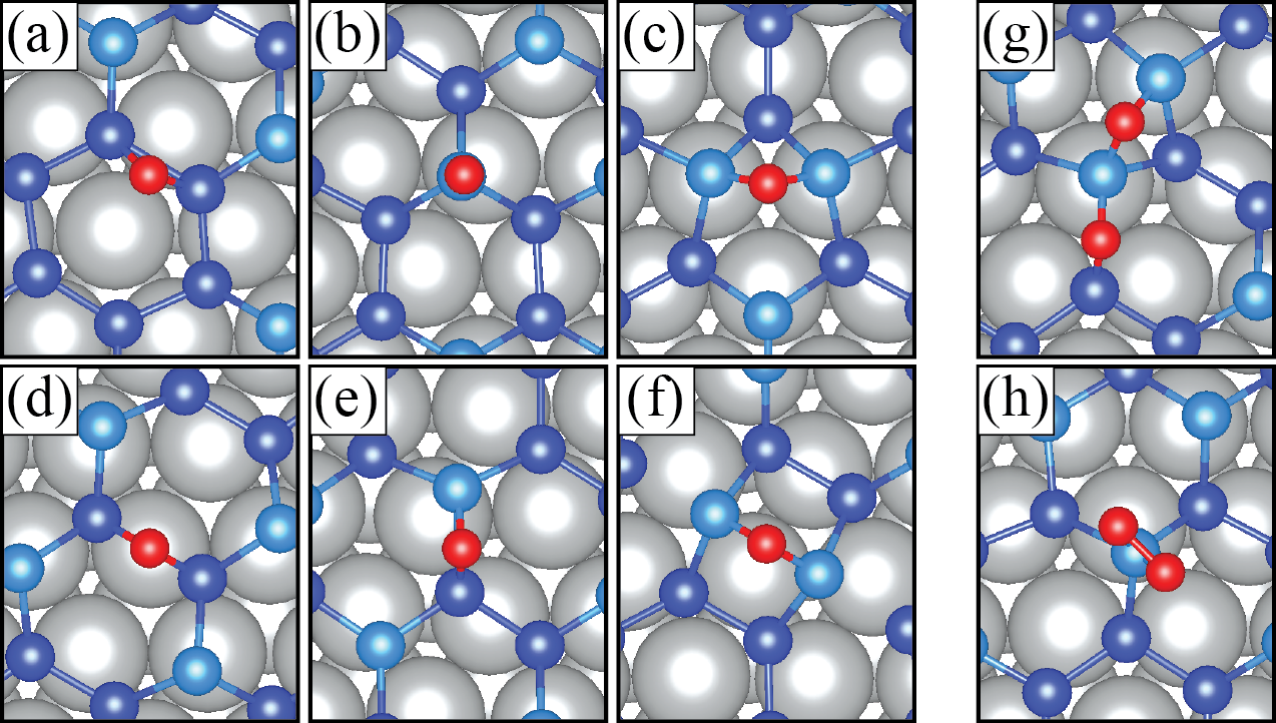
Final configurations of silicene-coated Ag(111) after capturing an oxygen atom at the sites (a) H_1_, (b) T_1_, (c) H_2_, (d) T_2_, (e) T_3_ and (f) H_3_. Final configurations (g) in-S and (h) top-S of silicene-coated Ag(111) after capturing an O_2_ molecule.

Silicene captures oxygen with a binding energy range of 5.8–6.5 eV. Compared to silver, silicene has a higher tendency to bind to oxygen. Figure 4 shows the oxygen–silicene bonding characteristics when the system reaches its local lowest energy. The least preferable sites for oxygen on silicene-coated Ag(111) are the sites where the highest distortion occurs and where oxygen has a low interaction with silicon atoms, namely H_2_, H_3_ and T_1_. On the other hand, the sites with the lowest distortion are the most preferable sites for oxygen on silicene-coated Ag(111), namely T_2_, H_1_ and T_3_ in the order from the lowest to the highest binding energy. The final configurations of T_2_ and H_1_ with oxygen presence seem to be equivalent. Oxygen binds to a lower silicon atoms, but the difference lies in the fact that the upper silicon atoms in T_2_ allow for more freedom and oxygen can take a position between them. Whereas, six lower silicon atoms tend to retain their hexagonal configuration and the oxygen atom finds itself in an upper-site position between two silicon atoms. The absence of a silicon–silicon bond in the T_2_ configuration results in a difference of 180 meV. The highest binding energy is calculated for T_3_ site. In this configuration, the oxygen atom enters a top-site position between upper and a lower silicon atoms. In this position, the lowest distortion occurs in the system so that oxygen binds with a high energy and leads to formation of an energetically favorable final configuration.

In the case of the O_2_ molecule, there are two final configurations, “in-S” and “top-S”, to be formed at the most favorable oxygen-capture sites. Figure 4g and Figure 4h show the in-S and top-S configurations, respectively. It is shown that silicon and oxygen strongly interact with each other (Table 1). SiO_2_ is one of the most stable compounds in nature. Therefore, silicon tends to bind two oxygen atoms in the form of SiO_2_ as depicted in Figure 4h. However, the energetically favorable configuration is the one in which oxygen atoms take separate positions between the silicon atoms (Figure 4g). In contrast to the other configuration, the silicon atoms in silicene can pluck the O_2_ molecule and are allowed to diffuse in the system. This promises a good oxidation barrier for the silver surface.

### Oxidation of the Ag surface in the presence of silicene coating

To investigate the application of silicene as an oxidation barrier, indentation calculations with oxygen were performed. Since O atoms and O_2_ molecules strongly interact with silicene, the diffusion of O/O_2_ in the lateral direction is not possible and the hollow sites of the hexagonal lattice are the only possible sites for the penetration into the structure. Therefore, the hollow sites are considered for the indentation simulation. There are four different hollow sites in the silicene structure on Ag(111), as shown in Figure 3b. The sites are denoted as H_1_, H_2_, H_2_’ and H_3_. H_2_ and H_2_’ sites coincide to the fcc and hcp sites of the Ag(111) surface, respectively.

**Figure 5:**
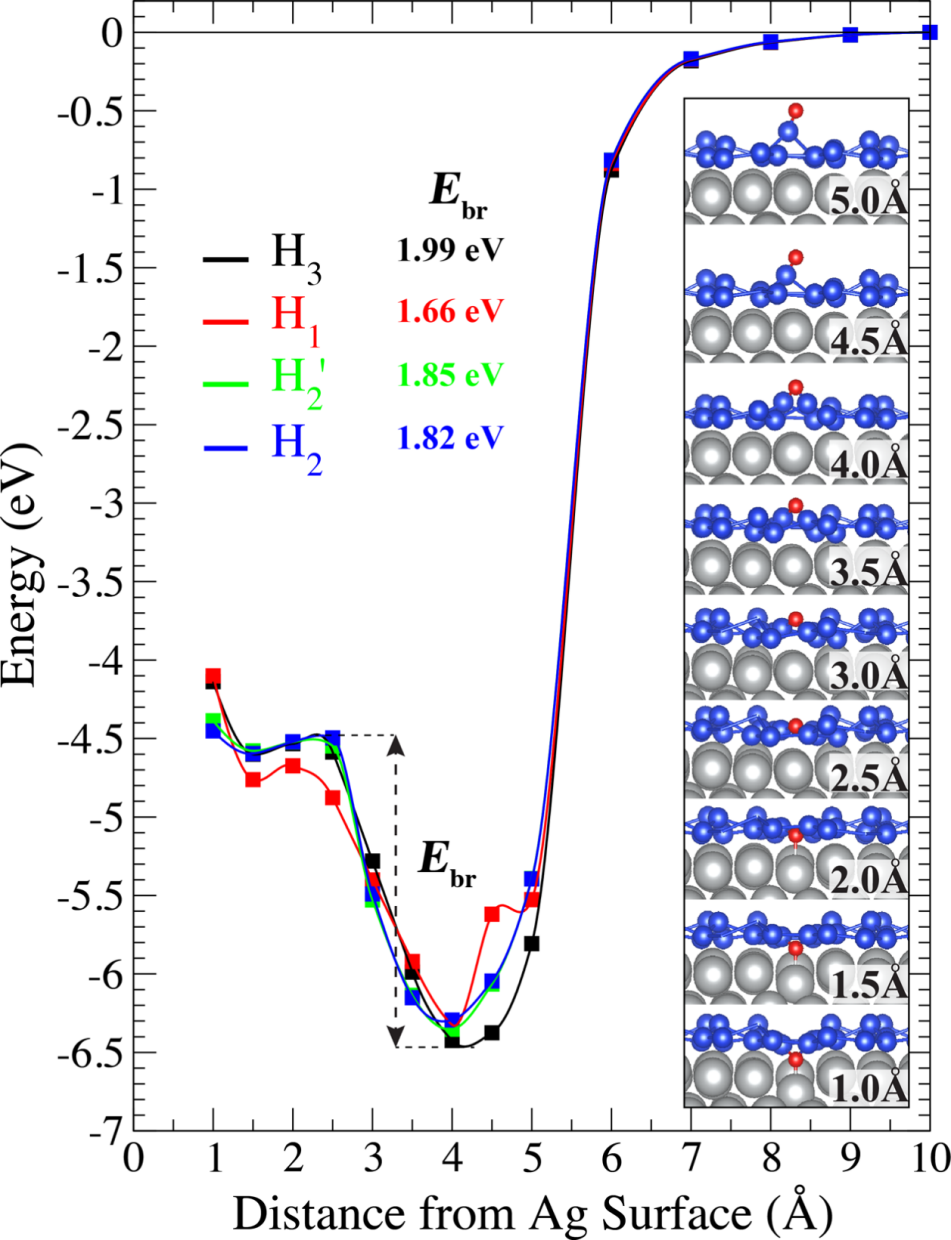
Indentation of an oxygen atom at different hollow sites. *E*_br_ is the energy barrier the oxygen atom needs to pass inside the silicene environment. Inset graph shows the oxygen progression in H_1_.

For the indentation calculations, a single oxygen atom is placed in the middle of a hollow site. Calculations are performed as follows: First, the oxygen atom is kept at a distance of about 10 Å from the Ag surface (ca. 8 Å to the silicene surface) and is approached to the surface in 1.0 Å steps. When the oxygen atom interacts with silicene, the step size is reduced to 0.5 Å. In Figure 5, the change of the total energy as a function of the vertical distance between oxygen atom and Ag surface is given for the different hollow sites. The barrier energies (*E*_br_), which are defined as the amount of energy needed for an oxygen atom to pass through a hollow site, are also shown in Figure 5. From lowest to highest, the values of *E*_br_ for H_1_, H_2_, H_2_’ and H_3_ are found to be 1.66, 1.82, 1.85 and 1.99 eV, respectively. The H_3_ hollow site has the highest energy barrier among all sites. At first, a fixed single oxygen atom attracts two upper silicon atoms to form one of the highly stable configurations. For that reason, the lowest ground-state energy occurs in this hollow site. While the single oxygen atom approaches the Ag surface, a barrier occurs up to ca. 2.5 Å where oxygen passes through hollow site exactly from upper site of silicene to the lower site of silicene. As seen in Figure 5, this also happens at the H_2_, H_2_’ and H_3_ hollow sites. H_2_ and H_2_’ are similar hollow sites. There is a small difference, ca. 30 meV, in energy barrier at these hollow sites. The lowest energy barrier, 1.66 eV, is seen in the H_1_ hollow site. Due to the planar structure of the silicon atoms at the H_1_ site, it is the most suitable hollow site for an oxygen atom to pass through silicene. Since the H_1_ hollow site has the lowest barrier energy, the side view of the structure for different distances is shown in the inset of Figure 5. At about 5 Å, the single oxygen atom pulls and binds one of the lower silicon atoms. Because further penetration of oxygen is not favorable at that distance from the Ag surface, a small local minimum occurs. Then, the other lower silicon atom binds the oxygen and the energy is lowered further. Due to the larger void size in the H_1_ hollow site than in the other hollow sites, it is easy to pass for the oxygen atom from the upper surface of silicene to the lower surface. At a distance of around 2.0 Å, the barrier has a different value than at the other hollow sites, because of the planar formation of the lower silicon atoms and also because of the larger void of the H_1_ hollow site.

**Figure 6:**
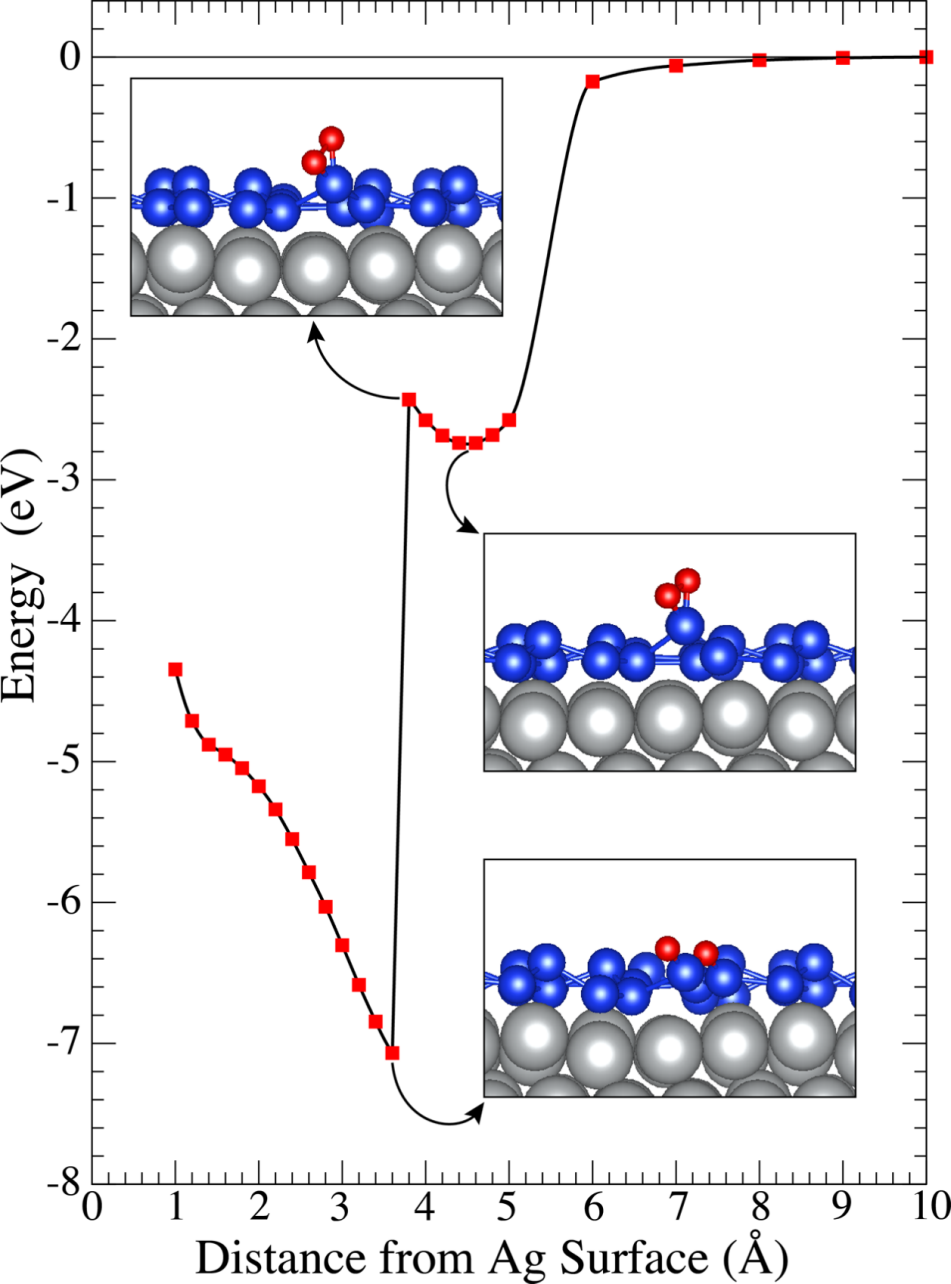
Indentation calculations for an O_2_ molecule. Inset graphs show structures according to local-minimum states of O_2_, before and after reaching the global minimum state of the system.

H_1_ is more permeable for oxygen atoms than other hollow sites. Hence, the indentation calculations for the O_2_ molecule are performed at the H_1_ site. One oxygen atom of the O_2_ molecule is fixed, the other non-restricted oxygen follows the fixed oxygen naturally. The oxygen molecule is moved closer to the Ag surface step by step from a distance of 10 Å via the fixed oxygen atom. The indentation process is shown with a small step size after the oxygen atom is captured by a silicon atom (Figure 6). There is a local-minimum state in which oxygen is still in the molecule form (see inset graphs in Figure 6), far from the Ag surface at a distance of about 4.5 Å. While the indentation of the O_2_ molecule continues, the energy increases up to some point. At distances below 3.8 Å, a sudden decrease in energy is seen. The inset graphs in Figure 6 show that the deterioration of the O_2_ molecule happens due to a strong interaction between oxygen and silicon atoms and after that silicene-coated silver system reaches a global-minimum state. The figure shows that the dissociation of the oxygen molecule is favorable and requires ca. 300 meV to exceed the energy barrier of transition. Since the H_1_ hollow site is the most permeable site, the maximum value of the transition energy barrier converges to ca. 300 meV. Two possible configurations, in-S and top-S (Figure 4g,h), are observed. The difference of those two minimum-energy states is similar (Table 1). In addition, oxidized silicene does not allow for a further indentation of a single oxygen atom. The locally forming silicon oxide structure attracts oxygen atoms more strongly. A local-minimum state as in FigureFigure 5 is not found between silicene and the Ag surface. Therefore, silicene becomes less permeable and more protective with increasing number of oxygen atoms. Our findings are consistent with the recent studies of oxidized silicene [28–29], which observed non-oxidized metal surfaces after the oxidation of silicene on metal substrates. One may claim that silicene retains its extreme reactivity to oxygen atoms even after forming localized silicon-oxide structures. As a result, silicene has great potential to capture unwanted atoms and to protect the metal surface.

## Conclusions

In this study, we performed first principles calculations to investigate the oxidation properties of α-silicene as a coating material on Ag(111). It was found that an O_2_ molecule interact with the Ag surface with a low binding energy, while a single oxygen atom interact strongly with the surface. The silicene coating on Ag surface was demonstrated as protective material from oxidation. In particular, large binding energies between a single oxygen atom and silicene were calculated for the possible adsorption sites. This strong interaction can break the oxygen–oxygen bond as well. Moreover, the energy barriers for the oxygen atom between silicene and Ag surface are quite high and sufficient for the protection of the metal surface. Indentation calculations of the O_2_ molecule showed that the molecule dissociates in the vicinity of silicene. It is also seen that an increase in oxygen atoms makes silicene more protective and silicene does not allow oxygen to pass to the metal surface. In conclusion, silicene has been proven itself as oxidation-resistant nanocoating material.
